# Effects of Prey-Mediated Sublethal Exposure to Imidacloprid and Nitenpyram on the Fitness and Predation Capacity in *Chrysopa pallens*

**DOI:** 10.3390/insects17020174

**Published:** 2026-02-05

**Authors:** Ting Chen, Shengwei Deng, Wei Wang, Ju Yao, Weifeng Guo, Yongsheng Yao

**Affiliations:** 1 College of Agriculture, Tarim University, Aral 843300, China; 13648130523@163.com (T.C.); 19197381379@163.com (S.D.); gwfzky@163.com (W.G.); 2 Institute of Plant Protection, Xinjiang Academy of Agricultural Sciences, Urumqi 830091, China; wlzforever2004@sina.com (W.W.); yaoju500@sohu.com (J.Y.)

**Keywords:** *Chrysopa pallens*, *Aphis gossypii*, sublethal effect, fecundity, functional response

## Abstract

*Chrysopa pallens* Stephens (Neuroptera: Chrysopidae) is a key predatory species in cotton agroecosystems. This study investigated the prey-mediated sublethal effects of imidacloprid and nitenpyram at low concentrations (LC_20_), on *C. pallens* when exposed via consumption of contaminated prey, assessing impacts on its development and predatory function. *C. pallens* is a key predatory species in cotton agroecosystems. This study investigated the prey-mediated sublethal effects of imidacloprid and nitenpyram (LC_20_) on the developmental performance and predatory capacity of *C. pallens*. Leaf-dipping bioassays were used to assess the toxicity of imidacloprid and nitenpyram to *Aphis gossypii* Glover (Hemiptera: Aphididae). Age-stage, two-sex life table analysis was conducted to evaluate their subsequent effects on the life history traits and predation performance of *C. pallens*. Imidacloprid was more toxic to *A. gossypii* than nitenpyram. Sublethal exposure marginally prolonged larval development, but the effect was not statistically significant. Both insecticides significantly extended the pupal stage, with nitenpyram inducing a greater delay. Imidacloprid markedly increased adult longevity, and both compounds significantly reduced female fecundity. Imidacloprid also suppress predatory behavior more potently, decreasing daily adult consumption and reducing first-instar attack rates by approximately 30%. Although all treatments followed a Holling type II functional response, both insecticides increased handling time and reduced searching efficiency. Overall, imidacloprid primarily inhibited predatory performance, whereas nitenpyram more strongly prolonged development and reduced critical population growth parameters. These findings provide essential evidence for ecological risk assessment and for refining the incorporation of natural enemies into cotton integrated pest management (IPM) strategies.

## Simple Summary

The control of cotton aphids, a major pest of cotton, often relies on insecticide applications. This practice, however, can adversely affect non-target beneficial insects that provide essential biological control services. Among these, the green lacewing (*Chrysoperla pallens*) is a key aphid predator whose role in maintaining field health may be compromised by such insecticide-mediated effects. This study investigated the effects of sublethal doses of imidacloprid and nitenpyram on green lacewings, mediated indirectly through the consumption of exposed aphids. Our results demonstrated that low-dose insecticide exposure mediated by aphids not only delayed larval development and reduced adult body size and fecundity, but also compromised the lacewings’ prey-capture efficiency. The two insecticides affected lacewings in different ways. Imidacloprid significantly compromised foraging efficiency, while nitenpyram more strongly suppressed population growth. This indicates that even non-lethal exposure can reduce the effectiveness of natural pest control. Incorporating these indirect pathways into risk assessments is a critical consideration for developing integrated pest management strategies that protect beneficial predators and promote more sustainable cotton systems.

## 1. Introduction

The cotton aphid, *Aphis gossypii* Glover (Hemiptera: Aphididae), is a globally significant piercing–sucking pest, recorded in more than 171 countries and infesting over 700 plant species [1]. It causes substantial annual losses through direct phloem feeding and indirect injury from honeydew deposition, which interferes with photosynthesis and reduces both cotton yield and fiber quality [2,3]. Although several environmentally friendly control tactics such as biological control [4,5] and plant resistance [6,7] are available, synthetic insecticides are still preferred during outbreaks for their rapid action and high efficacy [8,9]. However, the frequent application and overuse of chemical insecticides impose strong selection pressure on *A. gossypii* [10,11,12], promoting resistance development through mechanisms such as target-site mutations and enhanced detoxification metabolism [13]. This contributes to a self-reinforcing cycle of increased pesticide reliance, accelerated resistance evolution, and ecological disruption, which can negatively affect natural enemy communities and other non-target organisms [14,15].

*Chrysopa pallens* Stephens (Neuroptera: Chrysopidae) is a widely distributed predatory insect serving as an important biological control agent in both managed and natural ecosystems [16]. In the structurally complex cotton ecosystem, *C. pallens* serves as a key natural enemy of *A. gossypii*, exhibiting broad habitat adaptability and playing an irreplaceable role in integrated pest management (IPM) programs [17,18,19]. Both larval and adult stages prey on aphids, forming a close predator–prey association that contributes to natural suppression of aphid populations. A single *C. pallens* larva may consume approximately 1000 aphids, including about 870 *A. gossypii* individuals, with particularly high efficiency against early-instar aphids [20,21]. Even at low densities, *C. pallens* can effectively locate and attack aphids, thereby helping prevent population outbreaks. This regulatory capacity makes it a crucial natural enemy for suppressing cotton aphid populations and an important contributor to sustaining cotton yield and fiber quality [22].

The success of IPM depends strongly on the compatibility between chemical and biological control strategies [23,24,25]. However, a central challenge arises because many indispensable insecticides are non-selective, posing risks to beneficial arthropods and pollinators [26,27]. Reductions in natural enemy populations destabilize agroecosystems by weakening biological control, thereby triggering pest resurgence [28,29]. Consequently, comprehensive ecological risk assessments must extend beyond acute toxicity to include sublethal and chronic effects. Such impairments—including altered detoxification enzyme activity, disrupted development and reproduction, reduced longevity, and diminished foraging efficiency—can critically undermine the ecological performance of beneficial species [30]. These effects often emerge before mortality occurs and can accumulate over time, ultimately reducing the biological control services provided by natural enemies. This underscores the necessity of integrating comprehensive sublethal risk assessments into IPM frameworks.

Pesticide application is also a primary driver of resistance evolution in *A. gossypii* [31]. Repetitive exposure to insecticides accelerates the development of resistance, primarily through enhanced detoxification, target-site modifications, and reduced cuticular penetration, especially under excessive application rates or improper treatment intervals [32,33,34]. As resistance intensifies, pest managers often respond by increasing spray frequency and dosage to maintain efficacy. This creates a cycle that escalates ecological risks and amplifies adverse effects on natural enemies [35]. Sublethal exposure can alter key aspects of predator biology, including physiology, behavior, and population dynamics—effects which can manifest as changes in survival, fecundity, developmental rate, and predation efficiency. Therefore, integrated risk assessments must evaluate both lethal and sublethal effects [36].

In this study, we investigated the prey-mediated sublethal effects of two widely used neonicotinoids, imidacloprid and nitenpyram, against aphids, on *C. pallens* in cotton agroecosystems [37]. Imidacloprid is a systemic neonicotinoid insecticide effective against a wide range of sap-feeding pests, including aphids, thrips, and whiteflies, owing to its broad-spectrum activity and high systemic mobility [38,39,40,41]. Nitenpyram, a non-cyclic neonicotinoid with distinct target-site affinities and metabolic pathways, has been considered a potential alternative in rotation strategies, particularly where imidacloprid resistance has been documented [42]. Comparing these two neonicotinoids with differing chemical structures and modes of action can provide insight for facilitating rational insecticide rotation and enhancing natural enemy conservation in IPM strategies.

This study evaluated the trophically transferred effects of *A. gossypii* exposed to LC_20_ concentrations of insecticides on the development, survival, reproduction, and predatory performance of *C. pallens*. The assessment integrated demographic analysis (developmental duration, survival, fecundity) with behavioral evaluations (search efficiency and functional responses across larval instars). Our findings aim to elucidate the ecological consequences of such exposure, contribute to refined ecological risk assessments, and help design pesticide use strategies that are compatible with biological control in cotton IPM.

## 2. Materials and Methods

### 2.1. Insects and Chemicals

*A. gossypii* and *C. pallens* nymphs and adults (males and females) were collected from cotton experimental fields at Tarim University, Alar, Xinjiang (81°27′ E, 40°45′ N). *A. gossypii* colonies were maintained on cotton seedlings in laboratory conditions (25 ± 1 °C, 70 ± 5% RH, 16 h light: 8 h dark), with no prior exposure to pesticides. The same environmental conditions were maintained for all subsequent bioassays. The aphids were reared for more than 20 consecutive generations under these conditions. *C. pallens* were kept in insect rearing cages (33 cm × 33 cm × 33 cm) placed inside climate chambers (Model BIC-300, Shanghai Boxun Industrial Co., Ltd., Shanghai, China) under the same environmental regime. Lacewings were provided with fresh *A. gossypii* every 24 h.

Imidacloprid and nitenpyram of technical grade (Table 1) were initially dissolved in a small volume of acetone (Shanghai Macklin Biochemical Co., Ltd., Shanghai, China). After complete dissolution, serial dilutions were prepared using an aqueous solution of 0.05% Triton X-100 (Shanghai Macklin Biochemical Co., Ltd., Shanghai, China). The experiment used an acetone solution as the control treatment.

### 2.2. Toxicity Bioassay on Aphis gossypii

Insecticidal efficacy was evaluated using the leaf-dipping method. Technical grade pesticides were first dissolved in acetone to prepare stock solutions, which were then serially diluted with distilled water containing 0.05% Triton X-100 to generate 5–6 concentration gradients. The corresponding acetone–Triton solution served as the control treatment. Cotton leaves were immersed in each test solution for 10 s, air dried, and placed in Petri dishes lined with filter paper. Thirty healthy, uniformly sized aphids from laboratory colonies were transferred to each dish. The dishes were sealed with perforated plastic film to prevent escape while maintaining ventilation. Aphids were maintained under controlled conditions. Mortality was assessed after 24 h using a stereomicroscope. Individuals showing no movement in antennae or legs upon gentle probing with a soft brush were recorded as dead. Each treatment included four independent biological replicates, each consisting of a separate cohort of insects.

### 2.3. Prey-Mediated Effects of Sublethal Insecticide on the Development and Reproduction of Chrysopa pallens

Newly emerged adult *C. pallens* of both sexes were reared to sexual maturity and provided with healthy *A. gossypii* as a food source. Adults were maintained in cages under controlled conditions. White paper strips (100 × 35 mm) were introduced as oviposition substrates. After egg deposition, the strips were transferred to Petri dishes covered with gauze for protection and monitoring. Eggs were inspected at 2 h intervals to record precise hatching times.

Newly hatched first-instar larvae were placed individually in Petri dishes, with live *A. gossypii* provided that had been treated for 48 h to LC_20_ concentrations of imidacloprid or nitenpyram. A control group received untreated aphids. The prey was replenished every 24 h. Each treatment included three replicates with 20 larvae per replicate. Larval development was monitored daily, and developmental data for each instar were recorded. Upon adult emergence, body weight was measured. Adults were transferred to 1 L beakers for mating, with one pair per beaker. Ten mating pairs were randomly selected for detailed observation. Data were recorded every 8 h until all individuals died. Assessed parameters included mortality, adult longevity, oviposition latency, fecundity (total egg output), and egg hatch rate.

The age-stage, two-sex life table approach developed by Chi [43] was applied to analyze the experimental data. TWO SEX-MS Chart was used to compute different population parameters of *C. pallens* [44]. Demographic parameters calculated in this study, along with their formulas, are presented in Table 2. These included: age-stage specific survival rate (s_xj_), age-specific survival rate (l_x_), age-stage specific fecundity (f_xj_), age-specific fecundity (m_x_), age-specific maternity (l_x_m_x_), adult pre-oviposition period (APOP), age-stage life expectancy (e_xj_), and reproductive value (v_xj_). In addition, key population parameters, including the intrinsic rate of increase (r), finite rate of increase (λ), net reproductive rate (R_0_), and mean generation time (T), were estimated [45].

### 2.4. Prey-Mediated Effects of Sublethal Insecticide on the Predatory Capacity of Chrysopa pallens

Larvae of *C. pallens* at the first, second, and third instar were individually placed in Petri dishes and starved for 24 h to standardize hunger levels. Similarly, adult lacewings were held in 1 L beakers covered with mesh and deprived of food for 24 h prior to assays. *A. gossypii* were subjected to LC_20_ concentrations of imidacloprid or nitenpyram for 48 h, and the surviving aphids were utilized as prey. The larval prey densities were established as follows: 5, 10, 15, 20, and 25 aphids per dish for the first instar; 10, 20, 30, 40, and 50 for the second instar; and 30, 60, 90, 120, and 150 for the third instar. Adults received 100, 150, 200, 250, and 300 aphids in each beaker. All studies were performed under regulated conditions (25 ± 1 °C, 70 ± 5% RH, 16 h light: 8 h dark). A control group of untreated aphids was incorporated, consisting of five repetitions for each treatment. Predation was documented at 2, 4, 8, 12, and 24 h.

To measure the inhibition level of aphids treated with LC_20_ on *C. pallens* predation, we assessed the predation ratio of treatment groups compared to the control, where lower values indicate greater suppression. The formula for calculation is as follows:

$$\mathrm{Relative}\;\mathrm{Predation}\;\mathrm{Index}\;(\mathrm{RPI})\;=\;\frac{P_{t}}{P_{C}\;}$$

*P_t_*: predation on LC_20_-treated aphids

*Pc*: predation on untreated control aphids

### 2.5. Method for Determining the Functional Response

The predatory functional response of each developmental stage of *C. pallens* to *A. gossypii*, treated with sublethal concentrations of the two insecticides, was examined by fitting data to the Holling type II disk equation by nonlinear regression, in accordance with the methodology of Holling, C.S. et al. [33]. The equation is stated as follows:

$$N_{a}=\frac{a\;\times \;T\;\times \;N}{(1\;+\;a\;\times \;T_{h}\;\times \;N)}$$

*N_a_*: the number of *A. gossypii* consumed

*a*: the instantaneous attack rate

*N*: the initial density of aphids

*T*: the total experimental time (1 day in this study)

$T_{h}$: the handling time (time required for *C. pallens* to handle one aphid)

$\frac{1}{T_{h}}$: the maximum daily consumption capacity of *C. pallens*

The search efficiency of each developmental stage of *C. pallens* on the treated *A. gossypii* was evaluated using the equation given by Godfray, H.C.J. et al. [34]:

$$S=\frac{a}{(1\;+\;a\;\times \;T_{h}\;\times \;N)}$$

*S:* the Searching efficiency

*a*: the instantaneous attack rate

$T_{h}$: the handling time (time required for *C. pallens* to handle one aphid)

*N*: the initial density of aphids

### 2.6. Data Analysis

Dose–response relationships between insecticide concentration and aphid mortality were analyzed using probit analysis to estimate LC_20_ and LC_50_ values. Differences among treatments for life history traits (developmental duration, adult longevity, fecundity, and body weight) and population growth parameters were first tested for normality using the Shapiro–Wilk test. When the assumptions of normality were met, differences among treatments were analyzed by one-way analysis of variance (ANOVA), followed by Duncan’s multiple range test at a significance level of *p* < 0.05. Predation data, which involved count responses measured across different prey densities and exposure times, were analyzed using generalized linear mixed models (GLMMs). Life table parameters were calculated using the age-stage, two-sex life table approach implemented in the TWO SEX-MSChart program, and functional response parameters were estimated by nonlinear regression based on the Holling type II model. All statistical analyses were conducted using SPSS 26.0.

## 3. Results

### 3.1. Toxicity Bioassay of Aphis gossypii

The LC_20_ of imidacloprid against *A. gossypii* was determined to be 4.82 mg·L^−1^, significantly lower than that of nitenpyram (6.41 mg·L^−1^). *A. gossypii* demonstrated 1.33-fold greater tolerance to nitenpyram relative to imidacloprid. This trend was further accentuated at the LC_50_ level, where imidacloprid (12.34 mg·L^−1^) exhibited 1.89-fold higher toxicity to *A. gossypii* compared to nitenpyram (23.36 mg·L^−1^) (Table 3). Based on these results, the present study further evaluated the sublethal effects of imidacloprid and nitenpyram at their respective LC_20_ concentrations on the life table parameters and predatory behavior of *C. pallens*.

### 3.2. Prey-Mediated Effects of Sublethal Insecticide on the Developmental Duration of Chrysopa pallens

**Figure 1:**
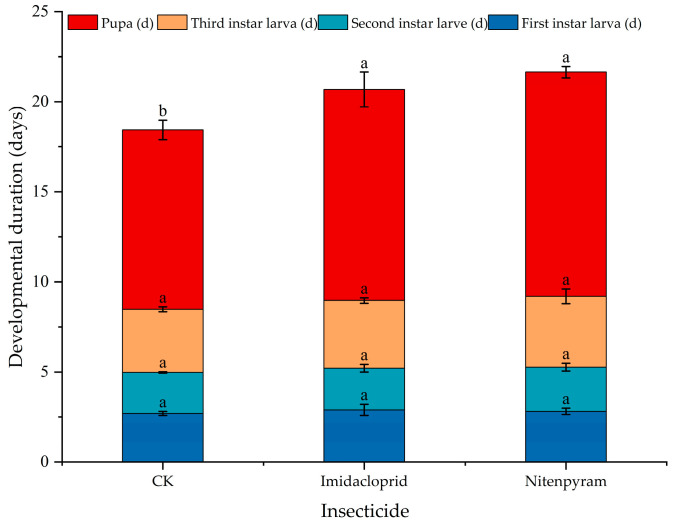
Effects of *A. gossypii* treated with sublethal concentration of two insecticides on developmental duration of *C. pallens.* Note: Data are presented as mean ± SE (*n* = 3 replicates with 20 individuals per replicate). Different colors represent different developmental stages. Statistical comparisons were performed among treatments within the same developmental stage (same color) using one-way ANOVA followed by Duncan’s multiple range test (*p* < 0.05). Different letters indicate significant differences among treatments.

Following consumption of aphids treated with LC_20_ of imidacloprid or nitenpyram, the developmental duration of all *C. pallens* stages was prolonged to varying degrees (Figure 1). Although the developmental duration of all larval instars was prolonged compared to the control, the differences were not statistically significant (*p* > 0.05). In contrast, both insecticides significantly extended the pupal period (*p* < 0.05). Based on the statistical results, the delaying effects of nitenpyram and imidacloprid on the pupal development of *C. pallens* under LC_20_ exposure were not statistically significantly different, although both insecticides showed a trend toward prolonged developmental duration.

### 3.3. Prey-Mediated Effects of Sublethal Insecticide on the Life History of Chrysopa pallnse

Adult *C. pallens* fed on aphids treated with LC_20_ concentrations of imidacloprid or nitenpyram exhibited marked alterations in life-history traits relative to the control (Table 4). Imidacloprid significantly prolonged adult longevity to 49.85 days, representing a 10.15% increase over the control (45.26 days), whereas the moderate increase observed under nitenpyram (47.04 days; 3.9%) was not statistically significant. A similar pattern was observed in female longevity: imidacloprid extended female lifespan to 58.86 days (17.3% longer than the control), while nitenpyram produced a smaller, nonsignificant increase (53.47 days). In contrast, male longevity remained statistically unchanged across treatments.

Reproductive performance was adversely affected by both insecticides. The pre-oviposition period (APOP) was extended from 24.98 days in the control to 27.14 days under imidacloprid exposure (an increase of approximately 8.65%) and to 26.49 days under nitenpyram exposure (an increase of about 6.00%). Fecundity was also significantly reduced: females laid an average of 452.40 eggs in the imidacloprid group and 455.72 eggs in the nitenpyram group, representing declines of 13.9% and 13.3%, respectively, compared with the control (525.40 eggs). Egg hatchability followed a similar inhibitory trend, decreasing from 85% in the control to 72% under imidacloprid and 78% under nitenpyram.

Both insecticides also suppressed adult body mass. Female weight decreased from 17.62 mg in the control to 15.75 mg and 15.91 mg in the imidacloprid and nitenpyram treatments, respectively, while male weight declined from 17.60 mg to 15.89 mg and 15.93 mg. Collectively, these findings demonstrate that prey-mediated exposure to imidacloprid and nitenpyram impairs both reproductive capacity and physiological condition in *C. pallens*, with imidacloprid exhibiting relatively stronger effects on extending female longevity, prolonging the pre-oviposition period, and reducing egg hatchability.

### 3.4. Prey-Mediated Effects of Sublethal Insecticide on the Population Growth Parameters of Chrysopa pallens

#### 3.4.1. Effects on the Fecundity of *Chrysopa pallens*

Prey-mediated exposure to sublethal concentrations (LC_20_) of imidacloprid and nitenpyram adversely affected multiple critical population growth parameters of *C. pallens*, as determined through age-stage, two-sex life table analysis. The intrinsic rate of increase (*r_m_*) declined from 0.13 day^−1^ in the control to 0.11 day^−1^ in both insecticide treatments, corresponding to a reduction of 15.4%. The net reproductive rate (R_0_) was reduced by 18.3% in the imidacloprid treatment and by 34.2% in the nitenpyram treatment relative to the control (Table 5).

Although the differences in R_0_ were not statistically significant, the decline was more pronounced under nitenpyram exposure. In addition, both insecticides significantly prolonged the mean generation time (T), which increased by 6.3% (imidacloprid: 42.66 d) and 6.5% (nitenpyram: 42.72 d) compared to the control (40.12 d) (Table 4). The finite rate of increase (λ) was significantly reduced to 1.12 day^−1^ in the nitenpyram group, with a 3.45% decrease relative to the control (1.16 day^−1^), whereas the decrease observed in the imidacloprid group (1.13 day^−1^) was not statistically significant. (Table 4). Overall, these results indicate that both insecticides suppressed the intrinsic growth rate and prolonged the generation cycle of *C. pallens*, with nitenpyram exhibiting stronger adverse effects on net reproduction and population growth capacity.

#### 3.4.2. Effects on the Age-Stage Survival Value of Chrysopa pallens

**Figure 2:**
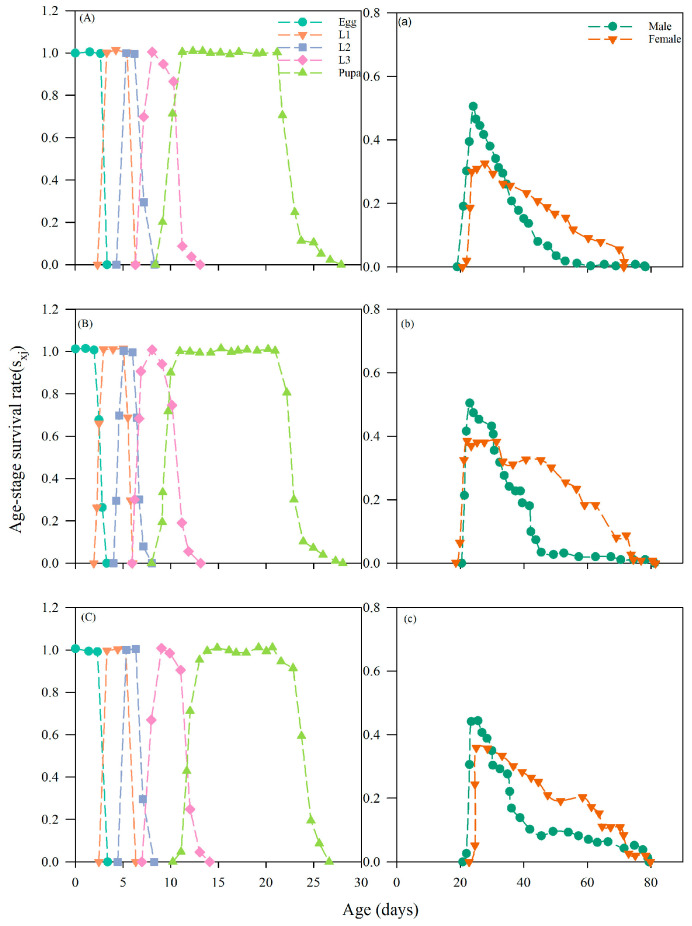
Effects of Feeding on *A. gossypii* treated with two insecticides at sublethal concentrations on the age-stage survival rate of *C. pallens.* Note: Life table analysis was based on 3 replicates with 20 individuals per replicate. (**A**,**a**) control group; (**B**,**b**) group feeding on *A. gossypii* treated with imidacloprid at LC_20_; (**C**,**c**) group feeding on *A. gossypii* treated with nitenpyram at LC_20_.

The age-stage survival rate (S_xj_) of *C. pallens* fed on untreated aphids or aphids treated with sublethal concentrations of imidacloprid or nitenpyram is presented. In the control group (Figure 2A), the S_xj_ surface showed high survival across juvenile stages and peak adult survival probabilities of 34% for females and 51% for males (Figure 2a). When larvae consumed aphids treated with imidacloprid at LC_20_ (Figure 2B), the overall shape of the survival trajectories remained similar, but maximum female survival increased slightly to 38%, while male survival was maintained at 51% (Figure 2b), indicating that prey-mediated imidacloprid exposure did not markedly depress adult survival. In contrast, feeding on nitenpyram-treated aphids (Figure 2C) resulted in a maximum female survival of 37.5% and a reduced male survival of 45.8% (Figure 2c), demonstrating a stronger inhibitory effect of nitenpyram on male survival. Across both insecticide treatments, the survival curves for juvenile stages were more extended along the age axis than in the control, which is consistent with the prolonged development described above and confirms that prey-mediated exposure to sublethal insecticides modifies both the level and timing of survival in *C. pallens.*

**Figure 3:**
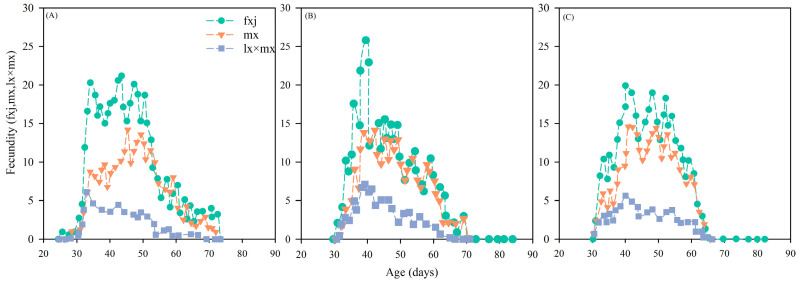
Effects of *A. gossypii* treated with sublethal concentration of two insecticides on female age-specific fecundity (f_xj_), age-specific fecundity (m_x_), and age specific maternity (l_x_ × m_x_) of *C. pallens.* Note: Life table analysis was based on 3 replicates with 20 individuals per replicate. (**A**) control group; (**B**) group feeding on *A. gossypii* treated with imidacloprid at LC_20_; (**C**) group feeding on *A. gossypii* treated with nitenpyram at LC_20_.

Evaluation of reproductive parameters, including age-stage-specific fecundity (f_xj_), age-specific fecundity (m_x_), and age-specific maternity (l_x_m_x_), revealed that consumption of aphids exposed to sublethal doses of imidacloprid and nitenpyram differentially affected the fecundity of *C. pallens* (Figure 3). This study revealed that prey-mediated exposure to sublethal concentrations of imidacloprid and nitenpyram altered the temporal pattern of fecundity in *C. pallens*. Analysis of the age-stage-specific fecundity (f_xj_) showed that the peak occurred on day 39 (f_xj_ = 25.81) (Figure 3B) in the imidacloprid LC_20_ group and on day 40 (f_xj_ = 19.92) (Figure 3C) in the nitenpyram LC_20_ group, which were 3 and 2 days earlier than the control (day 42; f_xj_ = 22.19) (Figure 3A), respectively. Notably, the imidacloprid treatment not only induced an earlier peak but also resulted in a higher maximum fecundity.

Regarding age-specific fecundity (m_x_), the nitenpyram LC_20_ treatment group attained the highest peak value of 14.39 (Figure 3C), marginally above the control value of 14.18, whilst the imidacloprid LC_20_ group recorded a value of 14.24 (Figure 3B), comparable to the control. Both insecticides significantly altered the timing of the m_x_ peak: the imidacloprid treatment group peaked 8 days earlier, while the nitenpyram group peaked 6 days earlier than the control (Figure 3C).

The fecundity curve of the nitenpyram LC_20_ treatment group closely mirrored that of the control, whereas the imidacloprid LC_20_ group displayed more substantial fluctuations and irregularities prior to day 50 (Figure 3B), suggesting a more complex effect of imidacloprid on the fecundity dynamics of *C. pallens*.

#### 3.4.3. Effects on the Age-Stage Reproductive Value of *Chrysopa pallens*

**Figure 4:**
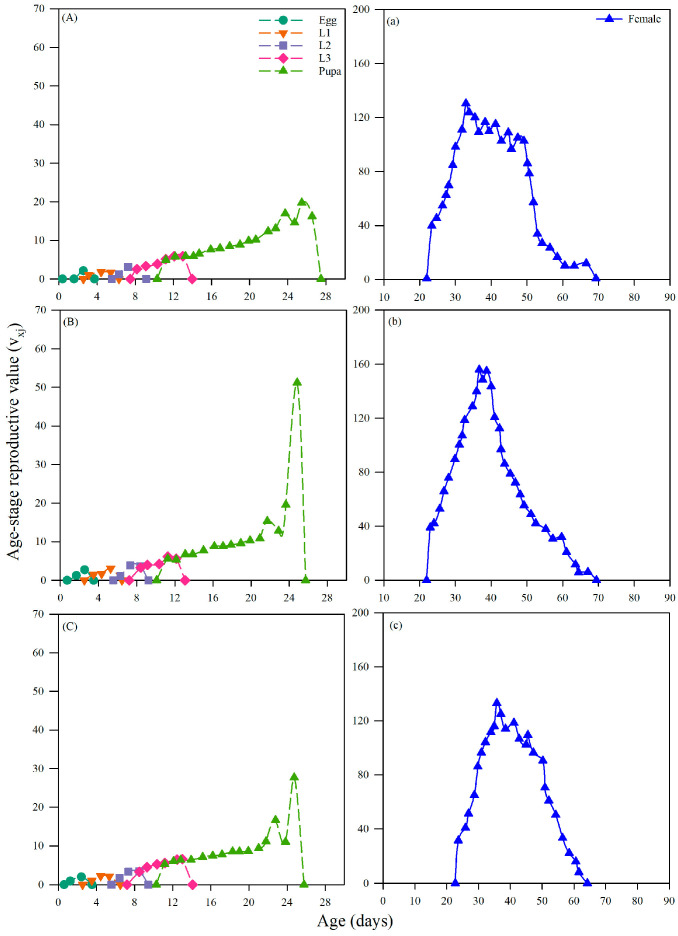
Effects of *A. gossypii* treated with sublethal concentration of two insecticides on female age-stage reproductive values (V_xj_) values of *C. pallens.* Note: Life table analysis was based on 3 replicates with 20 individuals per replicate. (**A**,**a**) control group; (**B**,**b**) group feeding on *A. gossypii* treated with imidacloprid at LC_20_; (**C**,**c**) group feeding on *A. gossypii* treated with nitenpyram at LC_20_.

The age-stage reproductive values (V_xj_) of *C. pallens* across the control and sublethal insecticide treatments. In the control group (Figure 4A), reproductive values increased rapidly after adult emergence, reaching an early and pronounced peak that reflects the normal reproductive schedule of healthy females. Exposure to imidacloprid (Figure 4B) substantially altered this pattern. Exposure to imidacloprid elevated the maximum age-stage reproductive value (V_xj_) of *C. pallens;* however, the timing of the peak was distinctly delayed, indicating that females required more time to accumulate sufficient resources for reproduction under insecticide stress. In contrast, nitenpyram exposure (Figure 4C) resulted in a peak reproductive value that was slightly lower than that of the control and also delayed to a later age, suggesting a more moderate but still evident inhibitory effect on reproductive performance. Together, these patterns demonstrate that both insecticides disrupt the temporal structure and magnitude of reproductive output in *C. pallens*, with imidacloprid causing a stronger shift in reproductive allocation than nitenpyram. Although the peaks of age-specific fecundity (f_xj_, m_x_) occurred earlier under both insecticides, the peak reproductive value (V_xj_) was delayed under imidacloprid. Although fecundity peaked earlier, the peak reproductive value (which also depends on survival schedule) occurred later. This apparent discrepancy arises because V_xj_ integrates both fecundity and age-specific survival; despite earlier egg production, delayed maturation and altered survival schedules shifted the timing of maximum reproductive value.

#### 3.4.4. Effects on the Age-Specific Life Expectancy of *Chrysopa pallens*

**Figure 5:**
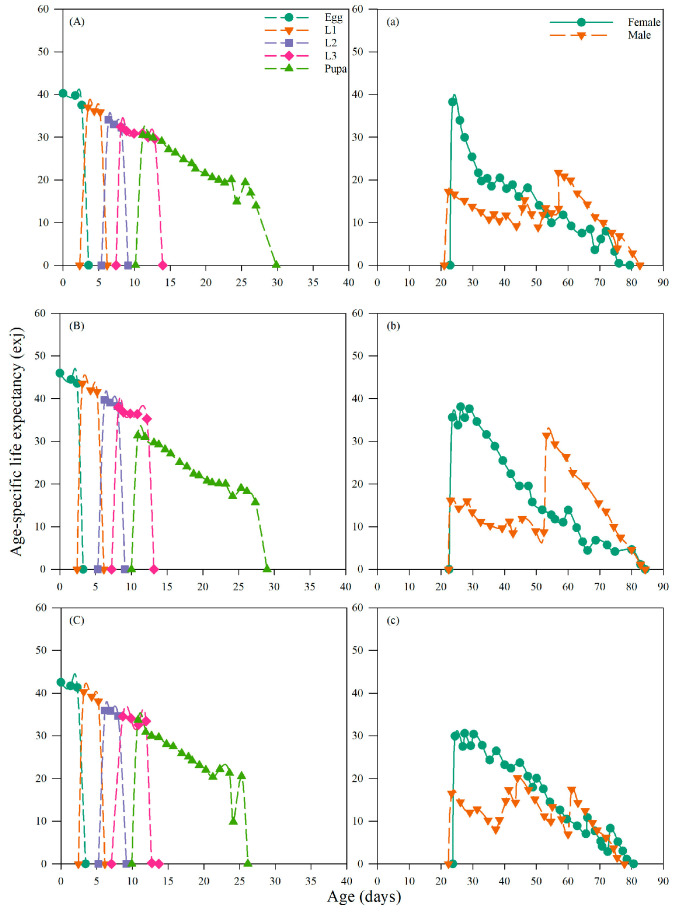
Effects of *A. gossypii* treated with sublethal concentration of two insecticides on age-specific life expectancy (e_xj_) of *C. pallens.* Note: Life table analysis was based on 3 replicates with 20 individuals per replicate. (**A**,**a**) control group; (**B**,**b**) group feeding on *A. gossypii* treated with imidacloprid at LC_20_; (**C**,**c**) group feeding on *A. gossypii* treated with nitenpyram at LC_20_.

The age-stage-specific life expectancy (e_xj_) of *C. pallens* across the control and the two insecticide treatments. In the control group (Figure 5A), life expectancy followed a typical declining pattern, with early instars exhibiting the highest expected remaining lifespan, which gradually decreased as individuals progressed through later stages and adulthood, ultimately corresponding to the shorter adult longevity observed in untreated lacewings. Dietary exposure to imidacloprid at LC_20_ (Figure 5B) altered this trajectory by elevating life expectancy across multiple developmental stages, consistent with the significant extension of adult longevity to 49.85 days. The increase was most evident during the adult period, where individuals maintained higher expected longevity throughout the reproductive phase, indicating a physiological shift toward survival maintenance under imidacloprid stress. In contrast, the nitenpyram LC_20_ treatment (Figure 5C) also extended life expectancy but to a lesser degree, in agreement with the more moderate increase in adult longevity to 47.04 days. The upward shift in e_xj_ under nitenpyram occurred across fewer stages and with reduced magnitude compared with imidacloprid, reflecting a milder impact on survival-related traits, despite its stronger suppression of population growth parameters such as R_0_ and λ. Taken together, the life expectancy curves demonstrate that sublethal insecticide exposure reshapes survival dynamics in *C. pallens*, with imidacloprid exerting the stronger prolonging effect and nitenpyram causing a moderate but still measurable extension.

#### 3.4.5. Effects on the Predation Rate of *Chrysopa pallens* at Different Time Intervals

**Figure 6:**
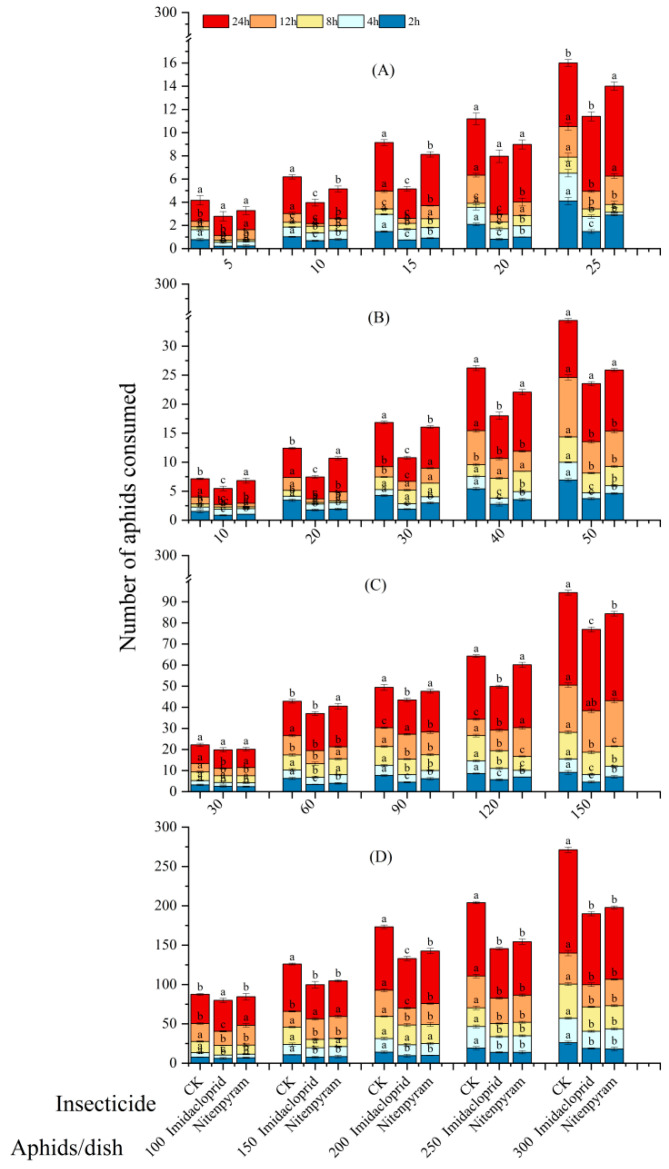
Effect of *A. gossypii* treated with two insecticides at sublethal concentrations on the predation by different developmental stages of *C. pallens.* Note: Predation denotes the number of aphids consumed (mean ± SE, *n* = 5). Different colors represent different treatments. Statistical comparisons were performed among treatments within the same color at each prey density and time point, and different letters indicate significant differences (GLMM, *p* < 0.05). (**A**) First-instar larvae at prey densities of 5–25 aphids. (**B**) Second-instar larvae at prey densities of 10–50 aphids. (**C**) Third-instar larvae at prey densities of 30–150 aphids. (**D**) Adults at prey densities of 100–300 aphids. Bars show cumulative predation over 2–24 h.

The predation capacity of *C. pallens* was significantly affected by aphid density, exposure duration, and the sublethal insecticides imidacloprid and nitenpyram. Moreover, the effects varied distinctly across the different developmental stages of the predator (Figure 6).

First-instar larvae showed the lowest predation capacity and highest sensitivity to insecticide exposure. At a density of 25 aphids per dish, consumption after 2 h was only 1.60 individuals in the imidacloprid group, significantly lower than that in the nitenpyram group (3.00 aphids) and the control (4.20 aphids), yielding an inhibition rate of 61.9% the highest across all stages (Figure 6A).

At a density of 40 aphids per dish, second-instar *C. pallens* larvae consumed 10.60 aphids over 12 h when fed aphids exposed to imidacloprid. This was lower than the consumption (11.80 aphids) recorded for larvae fed aphids exposed to nitenpyram. This result further confirms the more pronounced adverse effect induced by imidacloprid exposure. When prey density was increased to 50 aphids per dish, the suppressive effect of sublethal imidacloprid exposure on larval consumption remained statistically significant (Figure 6B).

Predation by third-instar *C. pallens* larvae was analyzed under a high prey density of 150 aphids per dish. During the 12 h observation period, larvae consumed 40.20 aphids treated with a sublethal concentration of imidacloprid and 43.40 aphids treated with nitenpyram. Consumption of imidacloprid-exposed aphids was significantly lower than that of aphids exposed to nitenpyram aphids (Figure 6C).

The predation capacity of *C. pallens* adults was compared under different prey densities. At a density of 100 cotton aphids per beaker, adults consumed 40.40 and 48.60 aphids within 12 h when fed aphids treated with sublethal concentrations of imidacloprid and nitenpyram, respectively. This represents a significant reduction in predation in the imidacloprid group compared to the nitenpyram group. In contrast, when prey density was increased to 300 aphids per beaker, consumption over 2 h reached 19.00 and 19.20 aphids for the imidacloprid and nitenpyram treatments, respectively, with no significant difference observed between them (Figure 6D).

Based on the above findings, it is evident that predation by different larval instars and adults of *C. pallens* was influenced by both prey density and sublethal insecticide exposure. Under consistent prey density, the negative impact of imidacloprid (at sublethal concentrations) on prey consumption became more pronounced across all larval instars and adults as the predation period shortened. Conversely, with a fixed predation duration, higher aphid densities led to a corresponding increase in the number of aphids consumed by both larvae and adults.

### 3.5. Prey-Mediated Effects of Sublethal Insecticide on the Functional Response of Chrysopa pallens

Based on the Holling type II functional response model, Table 6 summarizes the changes in predatory functional response parameters of *C. pallens* at different developmental stages when preying on *A. gossypii* exposed to sublethal concentrations (LC_20_) of imidacloprid and nitenpyram. Overall, both neonicotinoid insecticides significantly influenced the predator’s functional response, although the magnitude of inhibition varied with the insecticide type and the developmental stage of *C. pallens.*

The instantaneous attack rate (a) declined markedly across all developmental stages compared with the control, with imidacloprid exerting the strongest suppressive effect. The reduction was particularly pronounced in first-instar larvae, indicating that early instars are highly sensitive to sublethal insecticide exposure. While the attack rate generally reached higher levels in later stages (especially in adults) compared to early instars, it remained consistently lower than that of the control group, suggesting that sublethal exposure persistently impairs prey detection and attack efficiency.

Similarly, the handling time (T_h_) was significantly prolonged under both insecticide treatments. Increased handling time implies higher energetic and behavioral costs for completing each predation event, thereby reducing the effective predation frequency per unit time. The imidacloprid treatment produced a greater increase in T_h_ than nitenpyram, resulting in more severe inhibition of predatory efficiency.

As a combined consequence of the reduced attack rate and prolonged handling time, the maximum theoretical daily consumption (1/T_h_) was substantially lower in all developmental stages than in the control. For instance, the maximum daily consumption of first-instar larvae decreased from 27.78 individuals in the control to 19.53 individuals under imidacloprid exposure, whereas the reduction under nitenpyram was less pronounced. Although maximum consumption increased with developmental stage, neither insecticide treatment restored this parameter to control levels. Adults exhibited higher tolerance than larvae; however, imidacloprid still reduced their maximum consumption by more than 30%, demonstrating that mature predators remain susceptible to sublethal effects.

Comparative analysis further revealed that imidacloprid consistently imposed stronger inhibition on predatory performance than nitenpyram, as reflected by lower attack rates, longer handling times, and reduced maximum consumption. These findings indicate that the behavioral impacts of sublethal neonicotinoid exposure on *C. pallens* are distinctly insecticide-specific.

### 3.6. Prey-Mediated Effects of Sublethal Insecticide on the Foraging Efficiency of Chrysopa pallens

**Figure 7:**
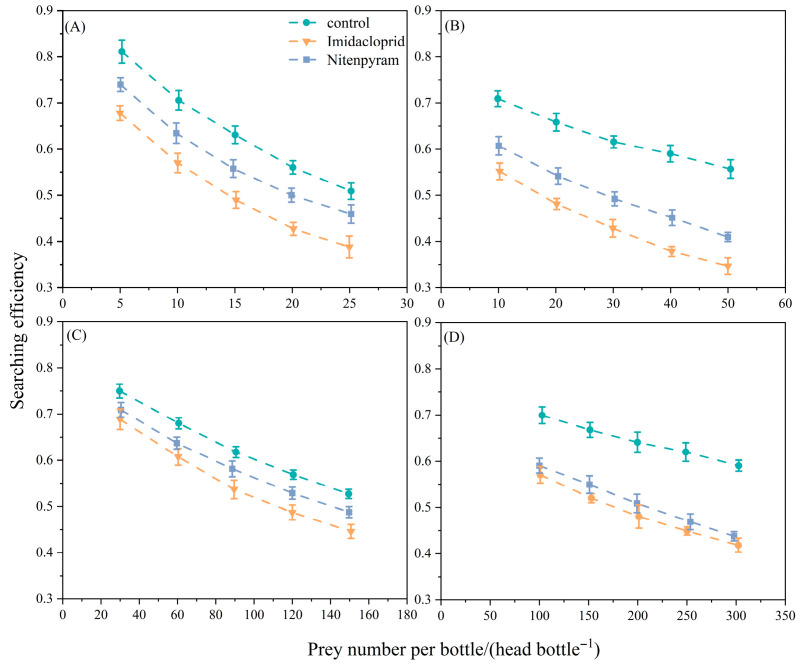
Effects of LC_20_-treated prey on the searching efficiency of different developmental stages of *C. pallens.* Note: Data are presented as mean ± SE (*n* = 5); (**A**) 1st instar larva; (**B**) 2nd instar larva; (**C**) 3rd instar larva; (**D**) Adult.

The searching efficiency of all developmental stages of *C. pallens*, including 1st, 2nd, and 3rd instar larvae and adults, declined with increasing aphid density when preying on aphids exposed to sublethal concentrations of imidacloprid or nitenpyram (Figure 7).

This pattern suggests that under high aphid densities, *C. pallens* requires more time to locate and capture individual prey. Compared with the control group, both insecticide treatments consistently reduced searching efficiency across developmental stages, indicating that aphids exposed to insecticides negatively influenced the foraging performance of the predator. Furthermore, searching efficiency was most strongly reduced under the imidacloprid treatment at all developmental stages, whereas the reduction observed under nitenpyram was less pronounced.

## 4. Discussion

Neonicotinoid insecticides are widely used in piercing–sucking pest management due to their high efficacy, strong systemic properties, and rapid action [46]. However, the long-term and frequent use of neonicotinoids in the field can result in their persistence in crops, pest populations, and surrounding environments, thereby raising concerns about their potential adverse effects on non-target beneficial arthropods [47]. Previous studies have shown that insecticides, while effectively controlling target pests, often have detrimental effects on key biological traits of natural enemies. These effects can arise through direct contact, ingestion of contaminated prey, or exposure to environmental residues, and include changes in development duration, survival and longevity, reproductive capacity, and predation efficiency [28,29,30,31,32,33,34,35,36,37,38,39,40,41,42,43,44,45,46,47,48,49,50].

Exposure to sublethal doses of insecticides via trophic transfer has received increasing attention, as it represents a key pathway leading to developmental and reproductive impairment in predatory natural enemies. In the present study, sublethal effects on growth and reproduction were observed in *C. pallens* after trophic exposure to LC_20_ imidacloprid and nitenpyram-treated aphids. These findings are consistent with previous reports indicating that sublethal exposure to imidacloprid, abamectin, and pymetrozine adversely affects development and fecundity in lacewings [51]. Specifically, both insecticide treatments significantly prolonged the adult pre-oviposition period (APOP); total fecundity and egg hatchability were markedly reduced (Table 4). The reduction in reproductive output is likely associated with sublethal disruption of behavioral and physiological regulatory processes. Insects rely heavily on pheromone-mediated communication for mating and reproduction [52]. However, sublethal pesticide stress can disrupt physiological state and behavior, thereby impairing mate recognition and courtship and ultimately reducing mating success [53,54]. Moreover, developmental delay and reduced adult body weight are commonly linked to endocrine disruption, prolonged metamorphosis, and altered energy allocation [55]. Previous studies indicate that insecticide effects on lacewings are often most pronounced at the pupal stage, as well as in traits related to body condition and emergence, rather than being limited to direct suppression of fecundity [56]. Consistent with this interpretation, the significant reduction in both female and male body weight under imidacloprid and nitenpyram treatments, together with the extended female longevity and reduced egg hatchability observed under imidacloprid exposure, suggests that sublethal neonicotinoid stress does not simply impair adult survival. Instead, it induces a reallocation of limited energy resources among growth, longevity, and reproduction, thereby delaying reproductive onset and reducing reproductive output [57].

At the population level, sublethal exposure to neonicotinoid insecticides significantly reduced the population growth potential and long-term persistence of *C. pallens*. Such effects can be directly reflected by changes in key population growth parameters. Life-table parameters, including the intrinsic rate of increase (r_m_), net reproductive rate (R_0_), finite rate of increase (λ), and mean generation time (T), are widely regarded as integrative indicators of population fitness and ecological risk in non-target organisms [58]. In the present study, sublethal doses of imidacloprid and nitenpyram significantly reduced r_m_, λ, and prolonged T in *C. pallens*. Although R_0_ did not differ significantly among treatments, a consistent downward trend was observed, indicating an overall suppression of population expansion capacity (Table 5). Comparable population-level responses have been reported in other natural enemies, with sublethal neonicotinoid exposure causing concurrent reductions in r_m_, R_0_, and λ, as well as delayed generation turnover in predatory lady beetles and mites [59,60]. Age-stage, two-sex life table analysis further showed that reduced population growth resulted primarily from delayed reproductive onset and diminished contributions of key reproductive stages. Sublethal treatments altered age-stage-specific fecundity (f_xj_), age-specific fecundity (m_x_), and reproductive value (V_xj_), leading to delayed reproductive peaks and an overall reduction in stage-specific contributions to population renewal (Figure 2, Figure 3, Figure 4 and Figure 5). Similar shifts in reproductive patterns have been observed in *C. pallens* following sublethal exposure to imidacloprid and thiamethoxam [61]. Overall, these results suggest that sublethal exposure to neonicotinoids may compromise the long-term persistence of natural enemy populations by disrupting key life-table parameters and age-stage reproductive structure.

Sublethal exposure to insecticides can adversely affect the predatory function of natural enemies by disrupting behavioral regulatory processes. Within this context of behavioral impairment, the present study showed that sublethal imidacloprid and nitenpyram reduced the searching efficiency and instantaneous attack rate (a) of *C. pallens* and prolonged handling time (T_h_) (Table 6). Similar suppressive effects on predation efficiency have been widely reported in other predatory natural enemies; for example, beta-cypermethrin and indoxacarb reduced prey consumption in *Chrysoperla sinica* [52], and imidacloprid reduced prey intake in *Harmonia axyridis* and *Propylea japonica* [62,63]. Moreover, in predatory species such as *Coccinella septempunctata* and *Orius laevigatus*, trophic exposure to aphids treated with sublethal concentrations of neonicotinoids similarly resulted in reduced predation rates, delayed feeding behavior, and impaired adult reproduction [64,65]. Insecticide-induced impairment of neuromotor coordination may account for the reduced attack rate, while disruption of feeding or metabolic processes likely underlies the prolonged handling time. Together, these effects reduce predation efficiency, despite the functional response conforming to Holling’s type II model [66]. Negative effects of sublethal insecticide exposure on searching ability have been documented in a wide range of insects. Methoxyfenozide significantly reduced host-searching efficiency in *Cydia pomonella* [67], while neonicotinoids such as imidacloprid impaired olfactory learning and memory in honey bees, resulting in reduced foraging efficiency [68,69]. Consistent with these findings, imidacloprid and nitenpyram reduced the searching efficiency of *C. pallens*, indicating that sublethal neonicotinoid exposure impairs key behavioral functions of predatory natural enemies.

Taken as a whole, our results show that sublethal neonicotinoid exposure has persistent adverse effects on reproduction, development, population dynamics, and predatory performance of *C. pallens* across multiple biological levels. Although such effects may not cause immediate mortality in natural enemies, their chronic accumulation across life stages and generations can progressively weaken biological control in integrated pest management (IPM) systems, without producing obvious ecosystem-level lethality. Therefore, assessments based solely on acute or lethal toxicity endpoints are insufficient to fully capture the ecological risks to beneficial arthropods. To better reconcile chemical and biological control in IPM programs, a more comprehensive framework is required-one that integrates both lethal and sublethal endpoints and incorporates key ecological traits of natural enemies, including fecundity, life-table parameters, and predatory performance.

## Figures and Tables

**Table 1:** Tested insecticides and concentrations.

Insecticides	Purity/%	Manufacturer	Location	Concentration Gradient/(mg·L^−1^)
				*Aphis gossypii*
Imidacloprid	95	Jiangsu Lüye Agrochemicals Co., Ltd.	Jiangsu, China	1.50, 3.00, 6.00, 9.00, 18.00
Nitenpyram	97	Jiangsu Lüye Agrochemicals Co., Ltd.	Jiangsu, China	2.19, 4.38, 8.75, 17.50, 35.00

**Table 2:** Main parameters and formulas used to calculate them.

Parameter	Abbreviation	Formula
Net reproductive	R_0_	R_0_ = $\sum_{x\;=\;0}^{\infty }l_{x}m_{x}$
Intrinsic rate of increase	r_m_	$\sum_{x=0}^{\infty }e^{-r(x\;+\;1)}l_{x}m_{x}$ = 1
Mean generation time	T	T = $\frac{\ln\left\{R_{0}\right\}}{r}$
Finite rate of increase	λ	λ =${\;e}^{r}$
Age-stage specific survival rate	s_xj_	s_xj_ = $\frac{n_{\mathrm{xj}}}{n_{01}}$
Age-stage life expectancy	e_xj_	e_xj_ = $\sum_{i\;=\;x}^{\infty }\sum_{y\;=\;j}^{\beta }s_{\mathrm{iy}}$
Reproductive value	v_xj_	v_xj_ = $\frac{1}{s_{\mathrm{xj}}}\sum_{i\;=\;x}^{\infty }e^{-r(i\;-\;x)}\sum_{y\;=\;j}^{\beta }s_{\mathrm{iy}}f_{\mathrm{iy}}$
Age-specific fecundity	m_x_	m_x_ = $\frac{\sum_{j\;=\;1}^{\beta }s_{\mathrm{xj}}f_{\mathrm{xj}}}{\sum_{j=1}^{\beta }s_{\mathrm{xj}}}$
Age-specific survival rate	l_x_	l_x_ = $\sum_{j\;=\;1}^{\beta }s_{\mathrm{xj}}$
Age-stage specific fecundity	f_xj_	f_xj_ = number of offspring produced by individuals at age x and stage j

**Table 3:** Toxicities of two insecticides against adults of *A. gossypii*.

Insecticides	Slope ± SE	LC_20_ (95% CL) /(mg·L^−1^)	LC_50_ (95% CL) /(mg·L^−1^)	Chi-Square	R^2^	(LC_50_) Index of Relative Toxicity
Imidacloprid	1.39 ± 0.10	4.82 (4.39–5.43)	12.34 (10.64–14.44)	9.31	0.984	1.89
Nitenpyram	0.96 ± 0.11	6.41 (5.27–8.17)	23.36 (18.61–29.47)	7.25	0.982	1.00

Note: Toxicities of two insecticides against adults of *A. gossypii* were evaluated 24 h after exposure. Each concentration was replicated four times, with 30 aphids per replicate. LC_20_ and LC_50_ values were estimated by probit analysis. Differences in toxicity were inferred based on the overlap of 95% confidence intervals (95% CL).

**Table 4:** Effects of *A. gossypii* treated with sublethal concentrations of two insecticides on the adult longevity, reproduction, and body weight of *C. pallens*.

Insecticides	Adult Longevity (d)	Female Longevity (d)	Male Longevity (d)	APOP (d)	Male Weight (mg)	Female Weight (mg)	Fecundity (Egg/Female)	Hatching Rate (%)
Control	45.26 ± 1.33 b	50.19 ± 2.16 b	40.32 ± 1.20 a	24.98 ± 0.22 b	17.60 ± 0.17 a	17.62 ± 0.65 a	525.40 ± 11.94 a	85 ± 1.00 a
Imidacloprid	49.85 ± 2.11 a	58.86 ± 3.63 a	40.84 ± 1.94 a	27.14 ± 0.80 a	15.89 ± 0.12 b	15.75 ± 0.60 b	452.40 ± 4.20 b	72 ± 2.00 c
Nitenpyram	47.04 ± 0.47 b	53.47 ± 3.19 ab	40.61 ± 2.44 a	26.49 ± 0.88 a	15.93 ± 0.10 b	15.91 ± 0.65 b	455.72 ± 14.96 b	78 ± 2.00 b

Note: Data are presented as mean ± SE (*n* = 3 replicates with 20 individuals per replicate). Different letters indicate significant differences among treatments based on one-way ANOVA followed by Duncan’s multiple range test (*p* < 0.05).; APOP, adult pre-reproductive period.

**Table 5:** Effects of *A. gossypii* treated with sublethal concentration of two insecticides on population growth parameters of *C. pallens*.

Parameters	Control	Imidacloprid	Nitenpyram
*R*_0_ (offspring/individual)	157.57 ± 33.44 a	128.71 ± 29.71 a	103.72 ± 30.56 a
*r_m_* (day^−1^)	0.13 ± 0.01 a	0.11 ± 0.03 b	0.11 ± 0.01 b
*T* (days)	40.12 ± 0.63 b	42.66 ± 0.73 a	42.72 ± 1.01 a
*λ* (day^−1^)	1.16 ± 0.02 a	1.13 ± 0.01 ab	1.12 ± 0.01 b

Note: Same as Table 4; *R*_0_, net reproductive rate (offspring/individual); *r_m_*, intrinsic rate of increase (day^−1^); *T*, mean generation time (days); *λ*, finite rate of increase (day^−1^). Means within a column with different letters are significantly different (*p* < 0.05, *n* = 3).

**Table 6:** Predation functional response and reaction parameters of *C. pallens* on *A. gossypii* treated with sublethal doses of two insecticides at different developmental stages.

Insect State	Insecticide	Functional Response Equation	Correlation Coefficient	Instant Attack Rate *a*	Handling Time *T_h_* (d)	Daily Maximum Predation Number 1/*T_h_*
1st instar larva	control	*N*a = 0.9517*N*/(1 + 0.0343*N*)	0.9626	0.9517	0.0360	27.78
	Imidacloprid	*N*a = 0.6624*N*/(1 + 0.0339*N*)	0.9456	0.6624	0.0512	19.53
	Nitenpyram	*N*a = 0.7752*N*/(1 + 0.0371*N*)	0.9841	0.7752	0.0479	20.88
2nd instar larva	control	*N*a = 0.7548*N*/(1 + 0.0069*N*)	0.9851	0.7548	0.0091	109.40
	Imidacloprid	*N*a= 0.6322*N*/(1 + 0.0074*N*)	0.9248	0.6322	0.0144	69.40
	Nitenpyram	*N*a = 0.6689*N*/(1 + 0.0091*N*)	0.9787	0.6689	0.0136	73.50
3rd instar larva	control	*N*a = 0.8324*N*/(1 + 0.0038*N*)	0.9830	0.8324	0.0046	218.80
	Imidacloprid	*N*a = 0.7546*N*/(1 + 0.0054*N*)	0.9556	0.7546	0.0072	139.70
	Nitenpyram	*N*a = 0.7765*N*/(1 + 0.0068*N*)	0.9465	0.7765	0.0088	114.20
Adult	control	*N*a = 0.8292*N*/(1 + 0.0031*N*)	0.9871	0.8292	0.0037	269.54
	Imidacloprid	*N*a = 0.6971*N*/(1 + 0.0037*N*)	0.9537	0.6971	0.0053	187.97
	Nitenpyram	*N*a = 0.7436*N*/(1 + 0.0033*N*)	0.9462	0.7436	0.0044	225.23

Note: *N*, initial prey density; *N*a, number of prey consumed; *a*, instantaneous attack rate; *T_h_*, handling time (days).

## Data Availability

The original contributions presented in this study are included in the article. Further inquiries can be directed to the corresponding author.

## Abbreviations

The following abbreviations are used in this manuscript:

ANOVA	Analysis of variance
APOP	Adult pre-oviposition period (adult pre-reproductive period)
a	Instantaneous attack rate
CK	Control (untreated group)
CL	Confidence limits (e.g., 95% CL)
e_xj_	Age-stage-specific life expectancy
f_xj_	Age-stage-specific fecundity
GLMM	Generalized linear mixed model
IPM	Integrated pest management
LC_20_/LC_50_	Lethal concentration causing 20%/50% mortality
m_x_	Age-specific fecundity
N	Initial prey density
N_a_	Number of prey consumed
R_0_	Net reproductive rate
RH	Relative humidity
rm	Intrinsic rate of increase
RPI	Relative predation index
R^2^	Coefficient of determination
S	Searching efficiency
SE	Standard error
S_xj_	Age-stage survival rate
T	Total experimental time (functional response)/mean generation time (life table)
T_h_	Handling time
V_xj_	Age-stage reproductive value
λ	Finite rate of increase
